# Metastatic Malignant Mesothelioma of the Tunica Vaginalis Testis Treated With Sequential Immunotherapy, Chemotherapy, and Radiotherapy With Prolonged Disease Stabilisation: A Case Report

**DOI:** 10.7759/cureus.114824

**Published:** 2026-08-20

**Authors:** Navin Mathiyalagan, Sarah Khan, Abigail Pascoe, Mitum Chauhan, Muhammad Adeel Sarwar, Wafaa N Abuzahra, Sahar Azad, Gaurav Mohindru

**Affiliations:** 1 Medical Oncology, Nottingham University Hospitals NHS Trust, Nottingham, GBR; 2 Radiation Oncology, Nottingham University Hospitals NHS Trust, Nottingham, GBR; 3 Histopathology, Nottingham University Hospitals NHS Trust, Nottingham, GBR; 4 Pathology, Nottingham University Hospitals NHS Trust, Nottingham, GBR

**Keywords:** chemotherapy, immune checkpoint inhibitors, malignant mesothelioma, palliative radiotherapy, tunica vaginalis

## Abstract

We report the case of a 77-year-old man with no known history of asbestos exposure who presented with a right-sided hydrocele. Histopathological analysis following hydrocele repair identified epithelioid-type malignant mesothelioma of the tunica vaginalis testis. Staging investigations confirmed disease confined to the hemiscrotum, and definitive management included right inguinal orchidectomy, excision of the spermatic cord to the deep inguinal ring, and wide local excision of the involved scrotal tissue.

Initial surveillance imaging showed no recurrence, but nodal metastatic disease subsequently developed. The patient received first-line immunotherapy with ipilimumab and nivolumab but experienced progressive nodal disease. Second-line carboplatin-pemetrexed chemotherapy resulted in a mixed radiological response after three cycles, with para-aortic nodal oligoprogression managed using palliative radiotherapy. Following palliative radiotherapy and three further cycles of carboplatin-pemetrexed chemotherapy, imaging demonstrated a partial radiological response.

Further surveillance revealed thoracic and intra-abdominal nodal progression, which was managed with a second course of palliative radiotherapy and was followed by renewed disease stabilisation. Subsequent progression involved pulmonary and intra-abdominal nodal disease, prompting initiation of third-line oral vinorelbine. Imaging demonstrated predominantly stable disease with isolated para-aortic oligoprogression, which was treated with a third course of palliative radiotherapy, after which vinorelbine was resumed. Approximately four years after diagnosis, the patient remained clinically stable, with an Eastern Cooperative Oncology Group (ECOG) performance status of 1, and was awaiting interval imaging to assess radiological disease status.

This case illustrates that an individualised sequential treatment strategy incorporating immunotherapy, chemotherapy, and palliative radiotherapy to sites of oligoprogression may be associated with periods of disease control in metastatic malignant mesothelioma of the tunica vaginalis testis.

## Introduction

Malignant mesothelioma of the tunica vaginalis testis (MTVT) is an exceptionally rare urological malignancy, accounting for less than 1% of all mesotheliomas [1-3]. It commonly presents as a hydrocele, scrotal swelling, or paratesticular mass and may initially mimic benign scrotal disease, resulting in delayed or incidental diagnosis [1-4]. Despite an apparently localised presentation, MTVT can exhibit an aggressive clinical course, with a propensity for recurrence and metastatic dissemination [1-3]. Evidence guiding the management of advanced or metastatic disease remains limited, with no established MTVT-specific systemic treatment strategy, and treatment approaches are largely extrapolated from malignant pleural mesothelioma [3].

This case contributes to the limited literature on metastatic MTVT by describing an individualised sequential multimodal treatment strategy incorporating immunotherapy, chemotherapy, and repeated courses of palliative radiotherapy to sites of oligoprogression, during which periods of disease control were observed.

## Case presentation

A 77-year-old man with an Eastern Cooperative Oncology Group (ECOG) performance status of 1 and no known history of asbestos exposure presented with a right-sided hydrocele. His past medical history was otherwise unremarkable, with no significant comorbidities.

**Figure 1:**
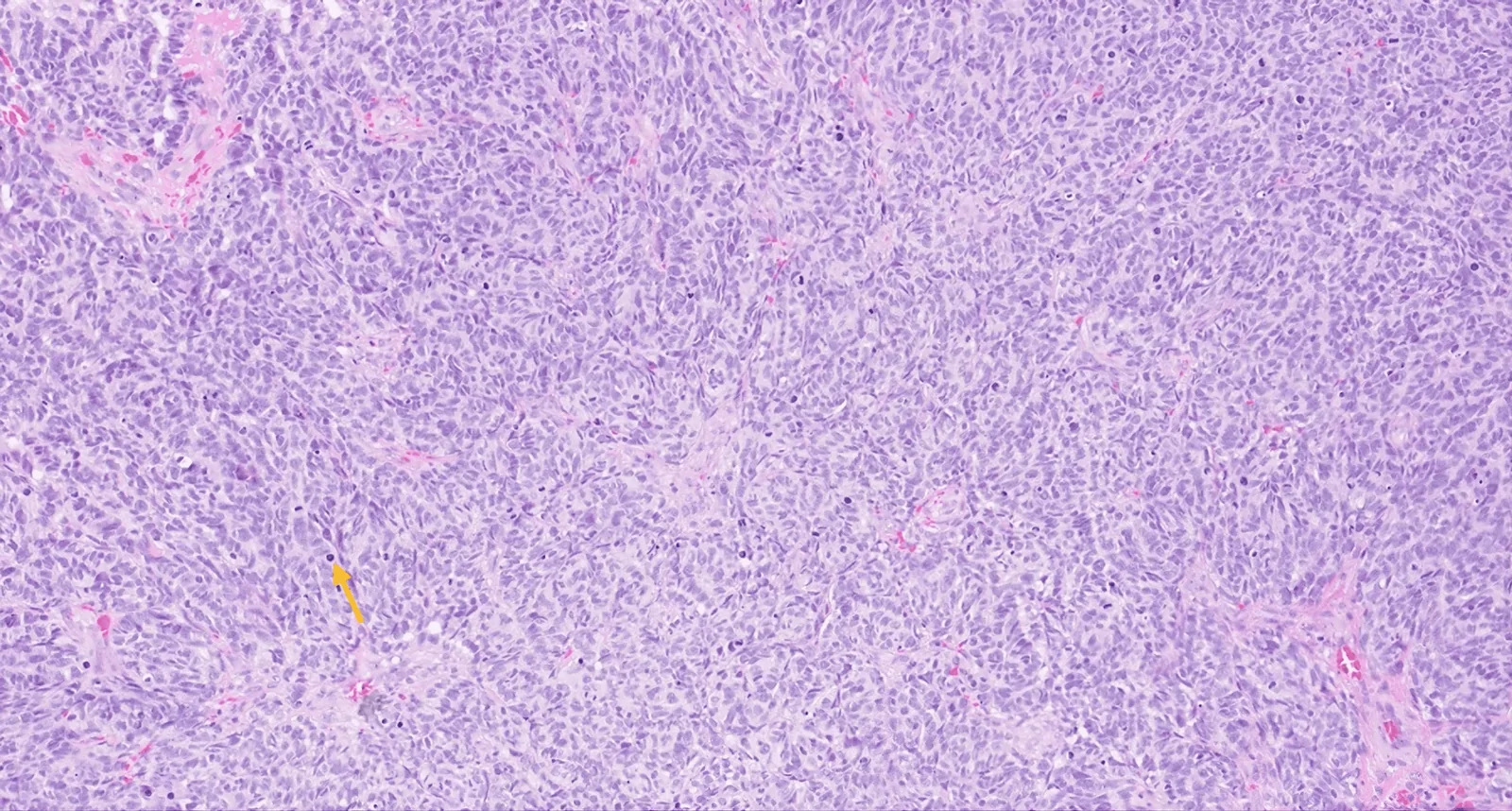
A high-power image of the tumour showing typical epithelioid morphology with numerous mitotic figures (yellow arrow).

**Figure 2:**
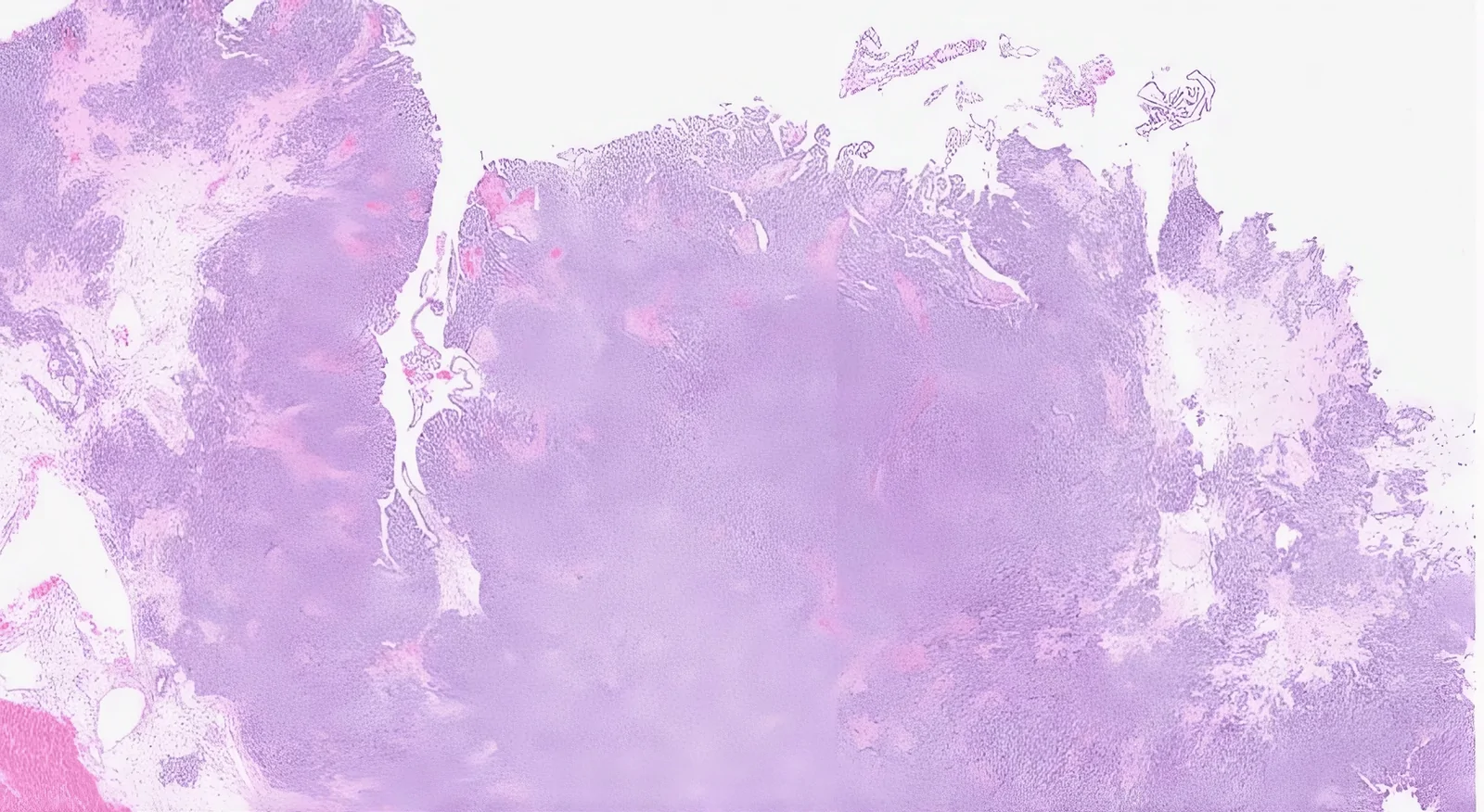
A low-power image of the tumour showing tubulopapillary and solid architecture.

**Figure 3:**
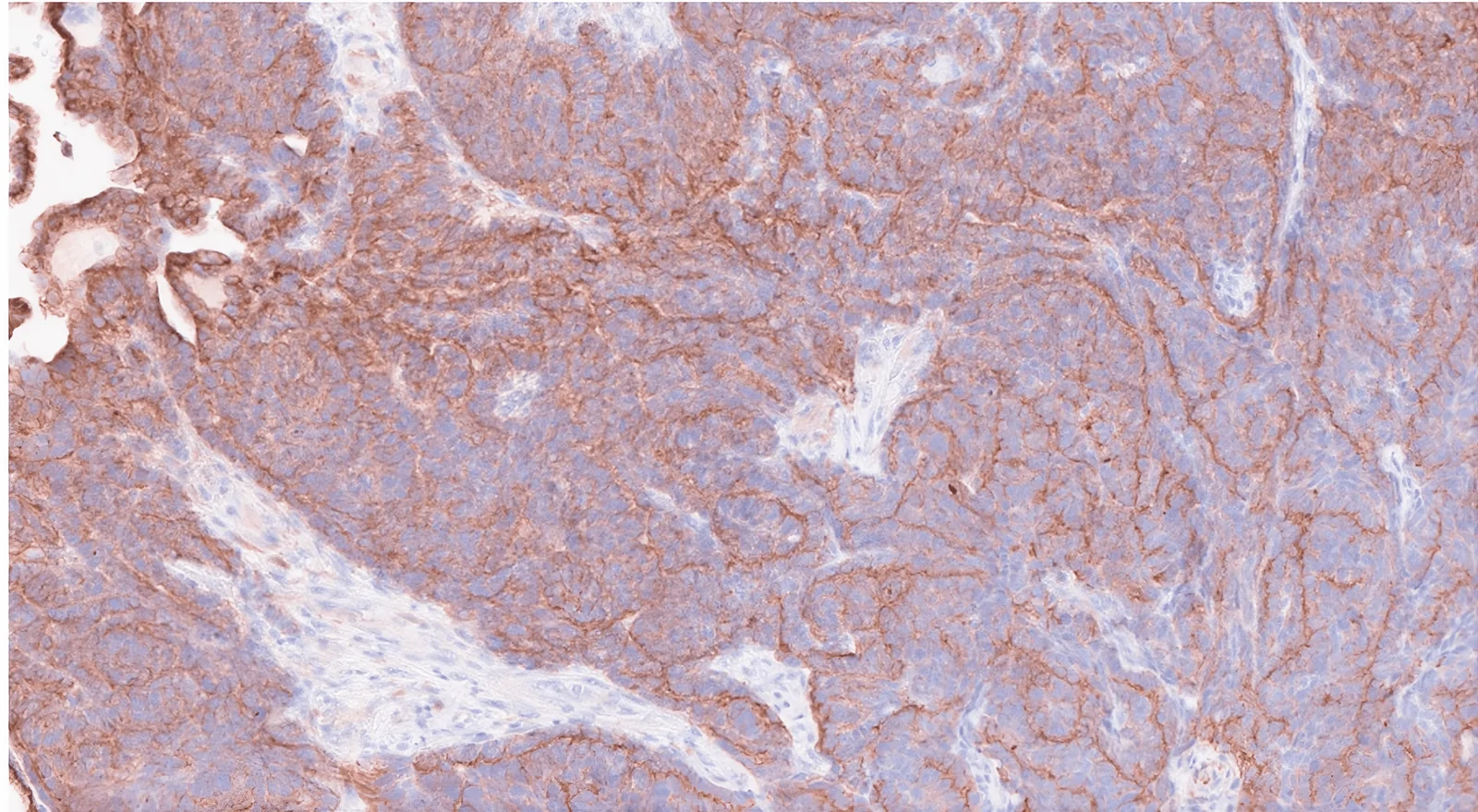
Immunohistochemistry demonstrating cytoplasmic positivity for pan-cytokeratin (AE1/AE3) (×20).

**Figure 4:**
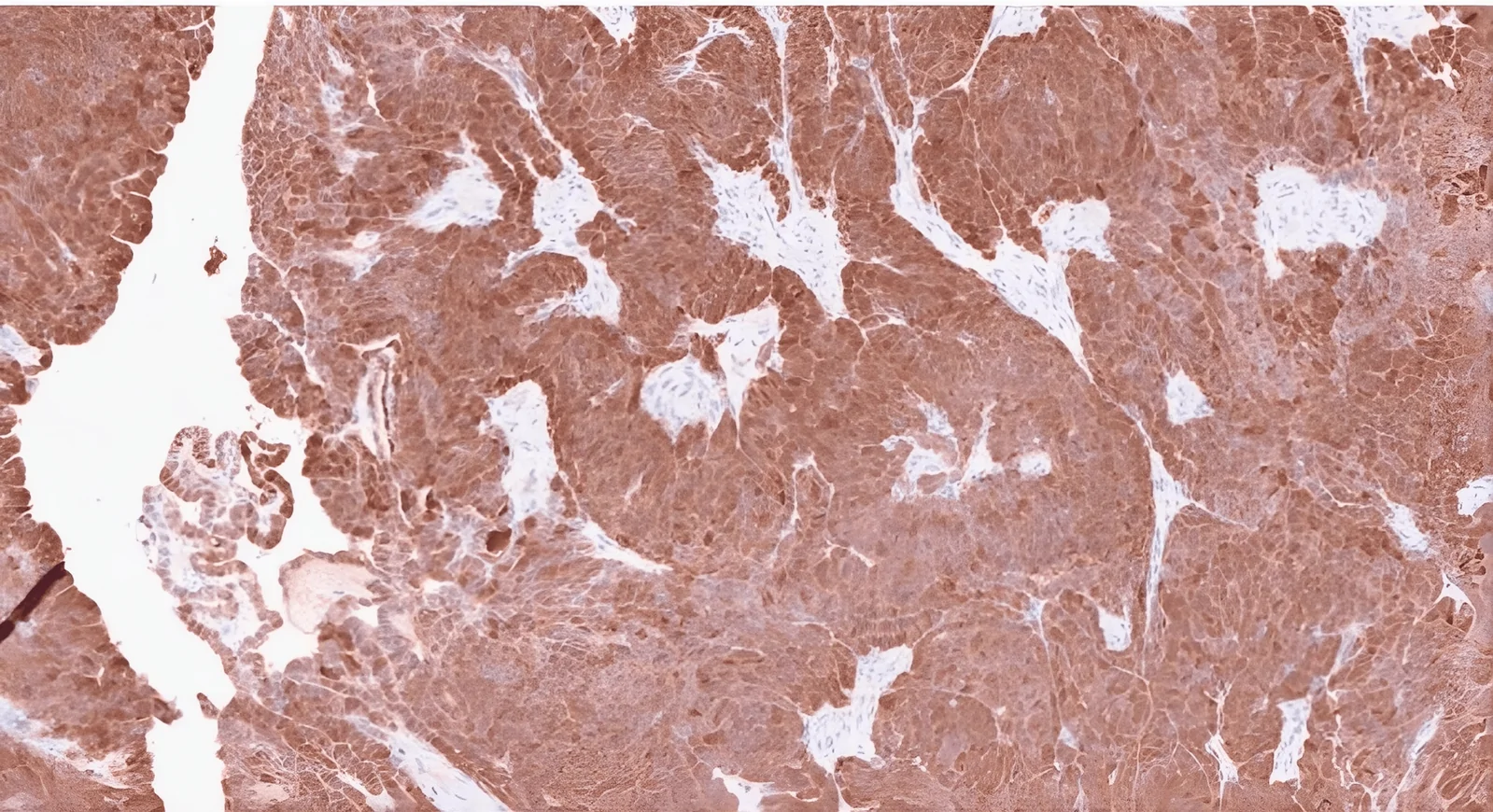
Immunohistochemistry demonstrating nuclear and cytoplasmic positivity for calretinin (×10).

**Figure 5:**
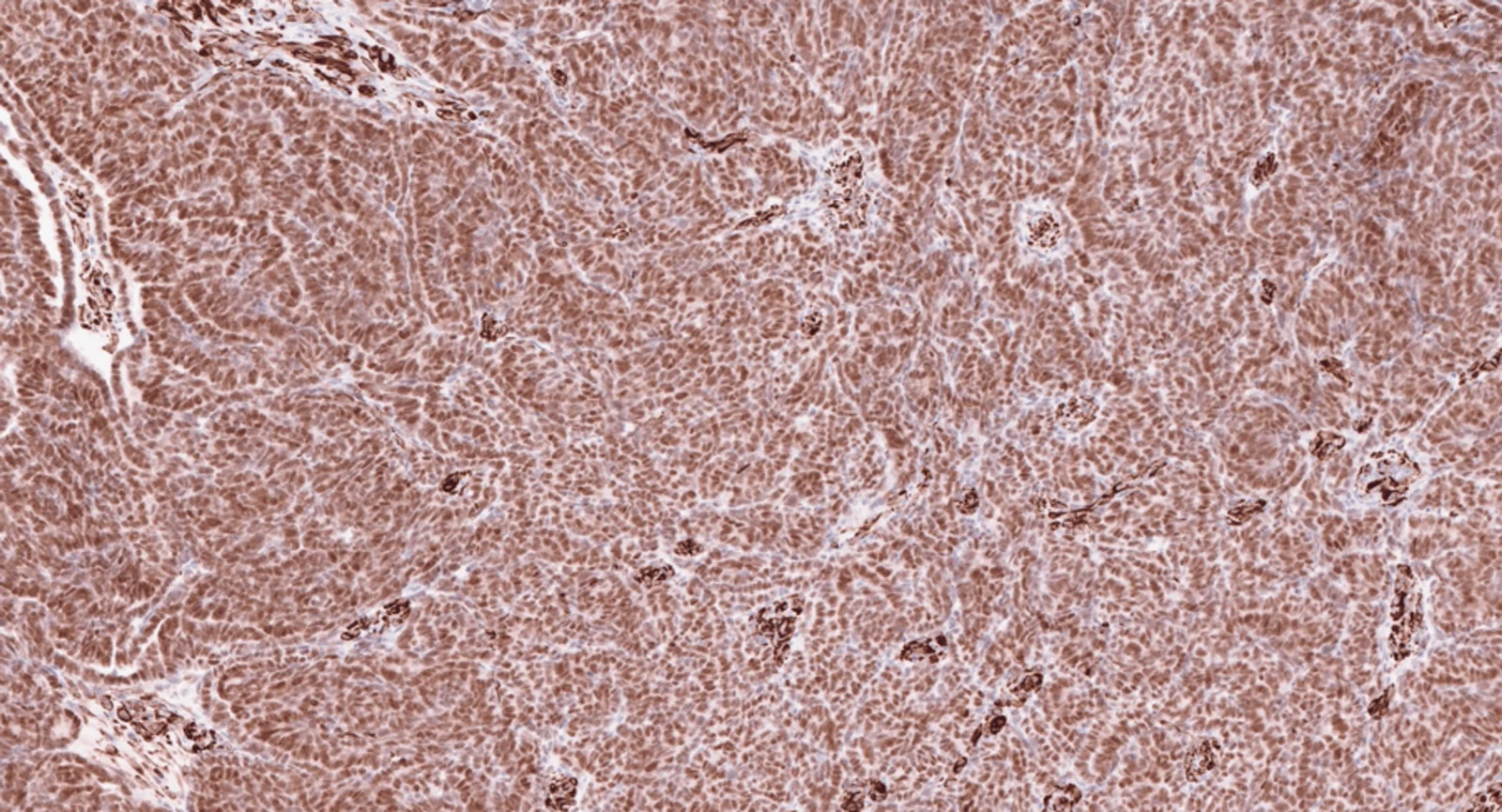
Immunohistochemistry demonstrating nuclear positivity for WT1 (×10).

He underwent right hydrocele repair in mid-Year 1. Intraoperatively, the surgeon observed abnormal tunica vaginalis tissue and performed a biopsy. Histopathological analysis confirmed epithelioid-type malignant mesothelioma of the tunica vaginalis with a predominant tubulopapillary growth pattern (Figures 1-2). Immunohistochemistry demonstrated cytoplasmic positivity for pan-cytokeratin (AE1/AE3), nuclear and cytoplasmic positivity for calretinin, and nuclear positivity for WT1 (Figures 3-5).

There was no staining for Ber-EP4, CD30, OCT3/4, PSA, AFP, or PSAP. p53 showed wild-type staining, while glypican-3 demonstrated only non-specific background staining. Histologically, the tumour demonstrated frequent mitotic figures, including atypical mitoses, together with multiple foci of stromal invasion. BAP1, MTAP, and CDKN2A testing were not performed, as these were not considered necessary by the regional mesothelioma multidisciplinary team (MDT). The overall morphological and immunophenotypic features were consistent with epithelioid malignant mesothelioma of the tunica vaginalis.

**Figure 6:**
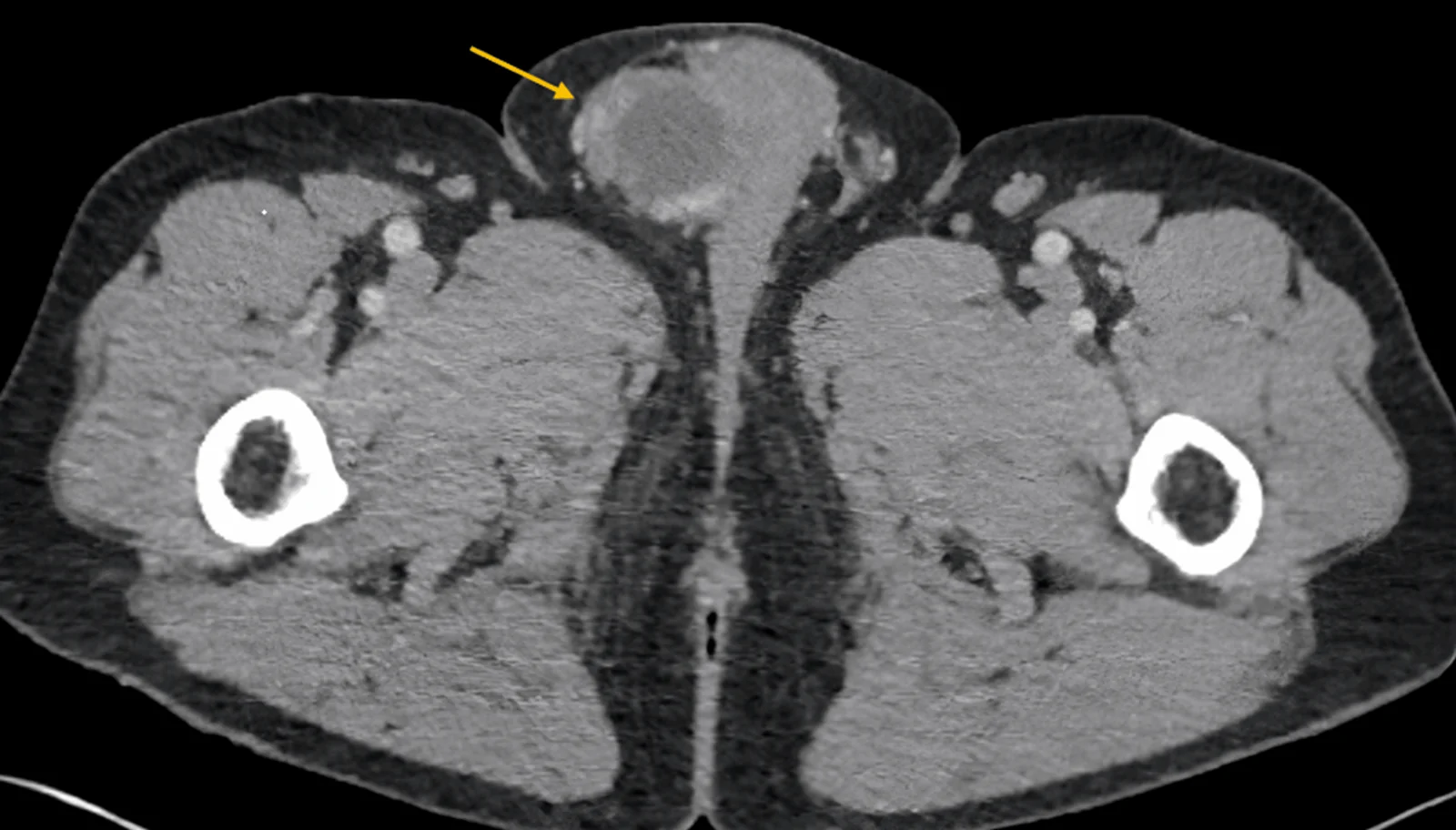
Contrast-enhanced axial CT image showing serpiginous enhancing soft tissue in the right hemiscrotum (yellow arrow), representing malignant mesothelioma of the tunica vaginalis.

**Figure 7:**
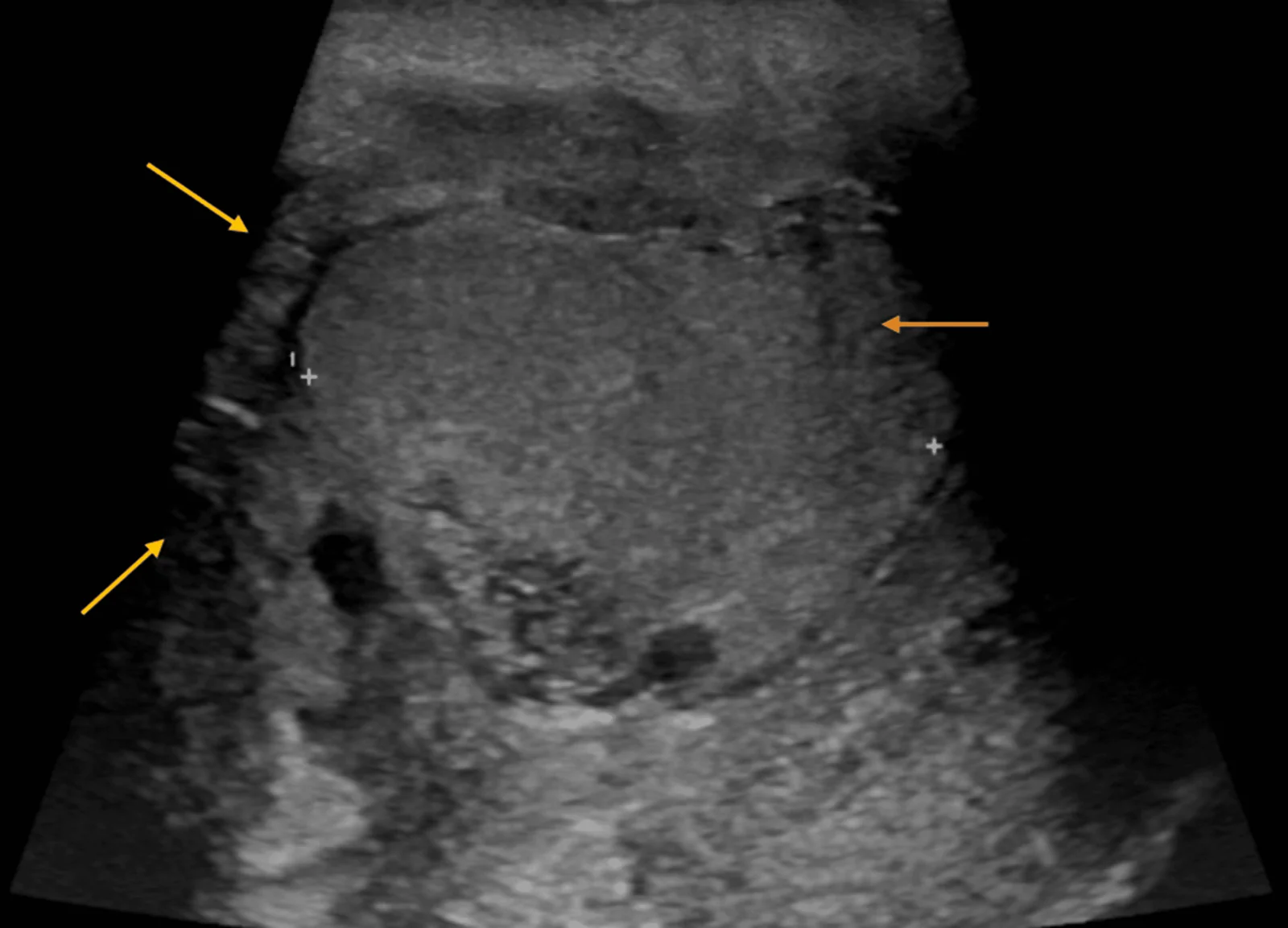
Ultrasound image of the right testis (orange arrow) showing multiple polypoid nodules arising from the tunica vaginalis (yellow arrows).

Staging CT of the thorax, abdomen, and pelvis performed in mid-Year 1 showed a small right hydrocele with enhancing serpiginous soft tissue in the superior lateral scrotum, corresponding to the known tunica vaginalis tumour, with no lymphadenopathy or distant metastases (Figure 6). Scrotal ultrasound demonstrated an enlarged right testis with a small cystic lesion and multiple vascular polypoid nodules arising from the tunica vaginalis in keeping with the known mesothelioma (Figure 7).

Based on these findings, the patient underwent right inguinal orchidectomy, excision of the spermatic cord to the deep inguinal ring, and wide local excision of the involved scrotal tissue, including the previous surgical site, to minimise the risk of tumour seeding. Histopathology confirmed epithelioid-type malignant mesothelioma of the tunica vaginalis. Owing to the rarity of this condition, the case was reviewed at the regional mesothelioma MDT, which recommended active surveillance. Whole-body PET-CT performed in late Year 1 demonstrated only postoperative changes, with no fluorodeoxyglucose (FDG)-avid disease.

**Figure 8:**
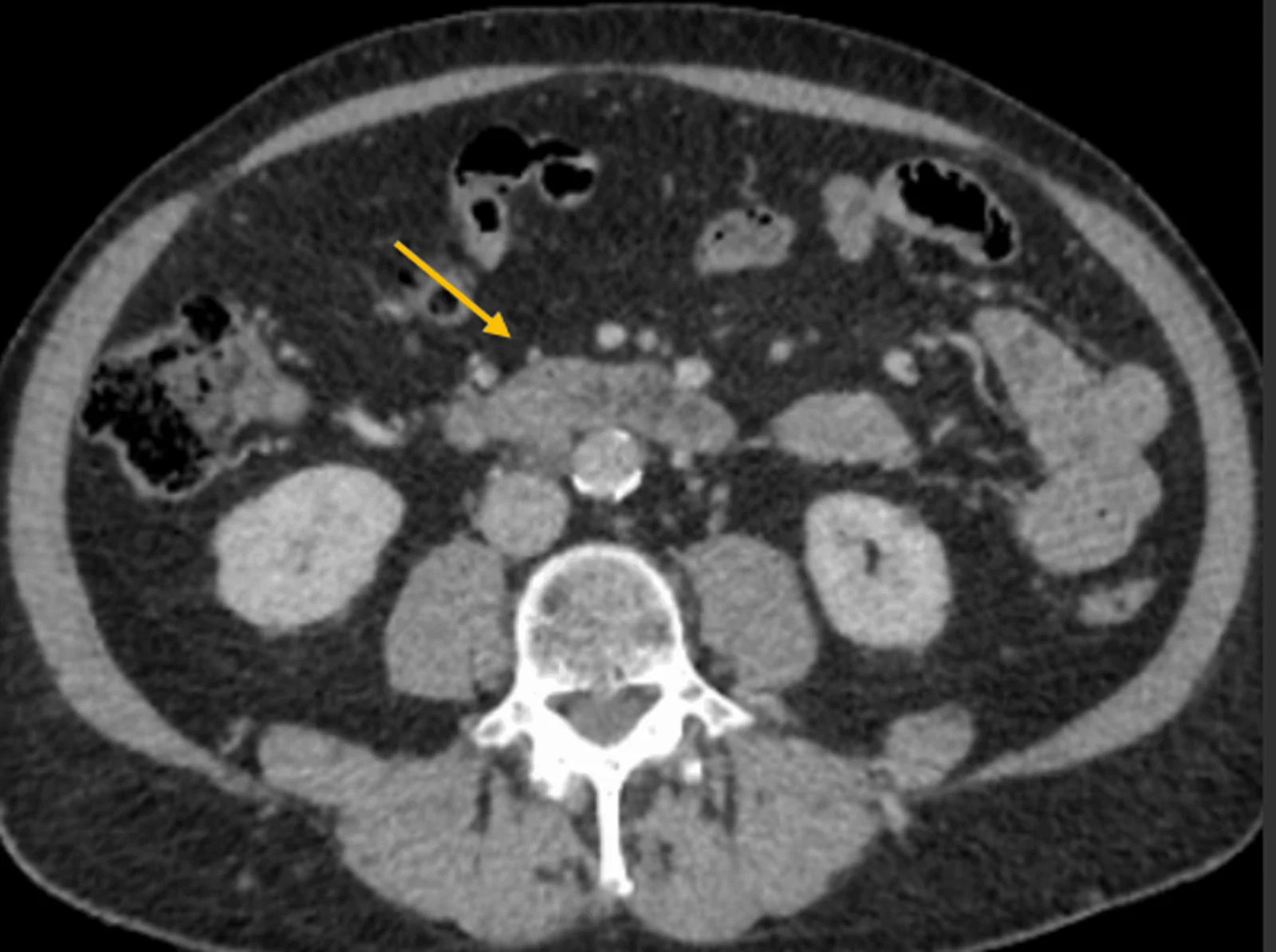
Contrast-enhanced axial CT image of the abdomen showing enlarged aortocaval lymphadenopathy (yellow arrow), considered consistent with metastatic disease following multidisciplinary team review.

The patient remained under close radiological surveillance. Follow-up CT scans performed in late Year 1 and early Year 2 demonstrated no evidence of recurrence. CT imaging performed in early Year 2 identified a new 9 mm aortocaval lymph node, which subsequently increased to 13 mm on repeat imaging in mid-Year 2 (Figure 8). The aortocaval lymph node was not biopsied; however, following MDT review of the serial imaging findings, the enlarging node was considered consistent with metastatic disease. Surgical resection was considered but deemed technically unfeasible, and the patient was referred to Medical Oncology.

**Figure 9:**
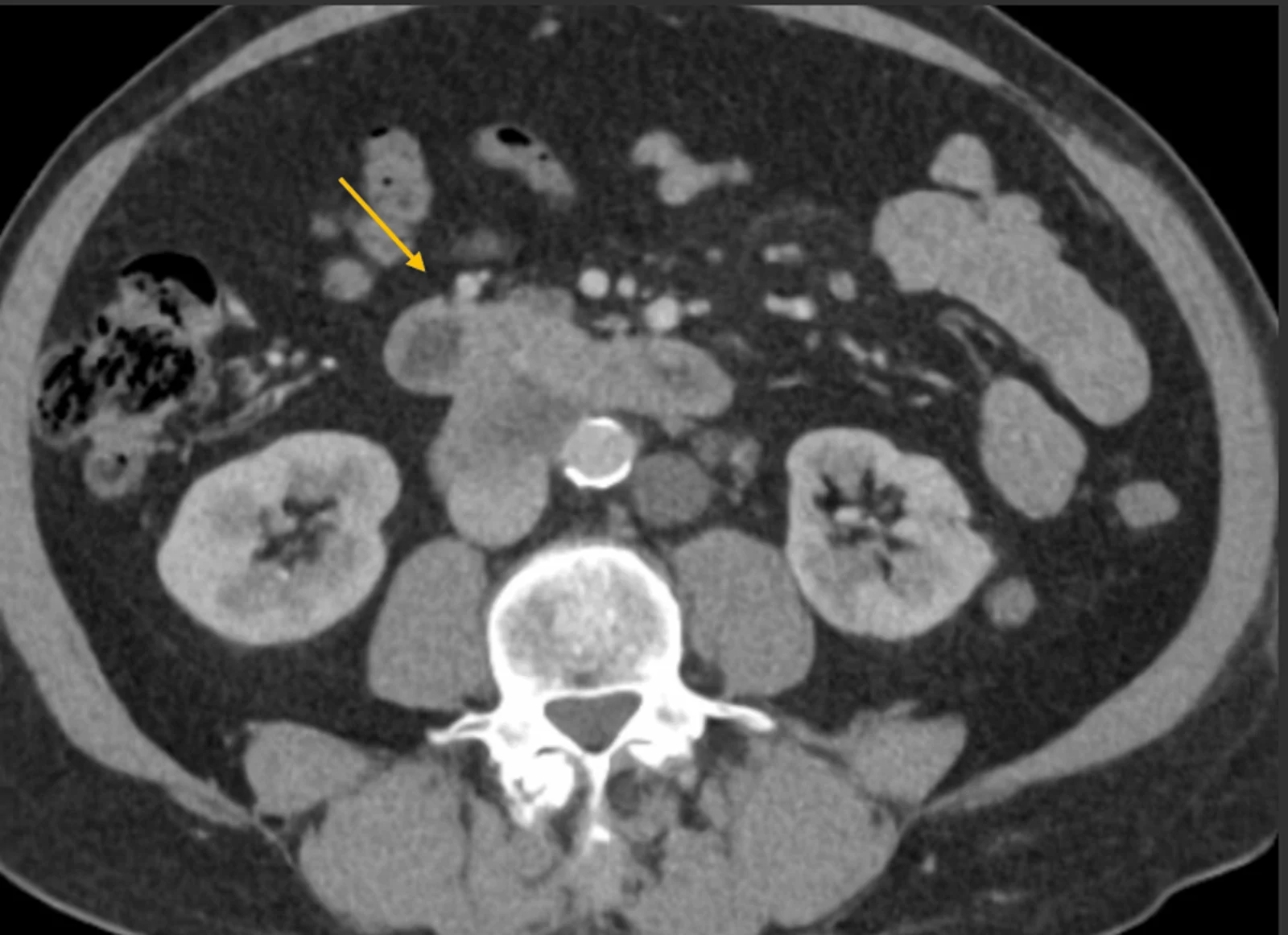
Contrast-enhanced axial CT image of the abdomen demonstrating progressive aortocaval lymphadenopathy (yellow arrow).

First-line immunotherapy with ipilimumab (1 mg/kg intravenously on Day 1) and nivolumab (360 mg intravenously on Days 1 and 22) commenced in late Year 2, with treatment repeated every six weeks. After four cycles, interim CT imaging in early Year 3 demonstrated disease progression, with continued enlargement of the aortocaval lymph node (Figure 9). Following review by the thoracic oncology MDT, immunotherapy was discontinued.

Second-line treatment with carboplatin (AUC 5 intravenously on Day 1) and pemetrexed (500 mg/m² intravenously on Day 1) commenced in early Year 3, administered every three weeks. After three cycles, interim imaging in mid-Year 3 demonstrated a mixed response, with reduction in the supradiaphragmatic lymphadenopathy, which had first been identified on CT imaging in early Year 3, but progression of the retroperitoneal para-aortic lymph nodes. Following MDT discussion, the patient received palliative radiotherapy (20 Gy in five fractions) to the bilateral retroperitoneal lymph nodes, followed by three further cycles of carboplatin-pemetrexed chemotherapy. Post-treatment imaging in mid-Year 3 demonstrated a partial response, with significant reduction in the para-aortic lymphadenopathy and stable disease elsewhere. The patient subsequently remained under close radiological surveillance. Imaging performed in late Year 3 showed further improvement, with reduction in size of the pathological lymph nodes within the left supraclavicular, right axillary, and para-aortic regions, indicating an ongoing response.

Radiological assessments of progression, mixed response, partial response, stable disease, and oligoprogression were based on contemporaneous formal radiology reports and MDT review. Formal application of RECIST 1.1 criteria was not consistently documented; therefore, response categories reflect the contemporaneously reported radiological assessments rather than retrospectively assigned RECIST-defined responses.

In early Year 4, imaging demonstrated further disease progression involving thoracic and intra-abdominal precaval and para-aortic lymph nodes. A second course of palliative radiotherapy (20 Gy in five fractions using an intensity-modulated radiotherapy (IMRT) technique to minimise toxicity) was delivered to the intra-abdominal lymph nodes, resulting in a reduction in nodal burden on imaging performed in mid-Year 4. Follow-up imaging in mid-Year 4 demonstrated stable disease.

**Figure 10:**
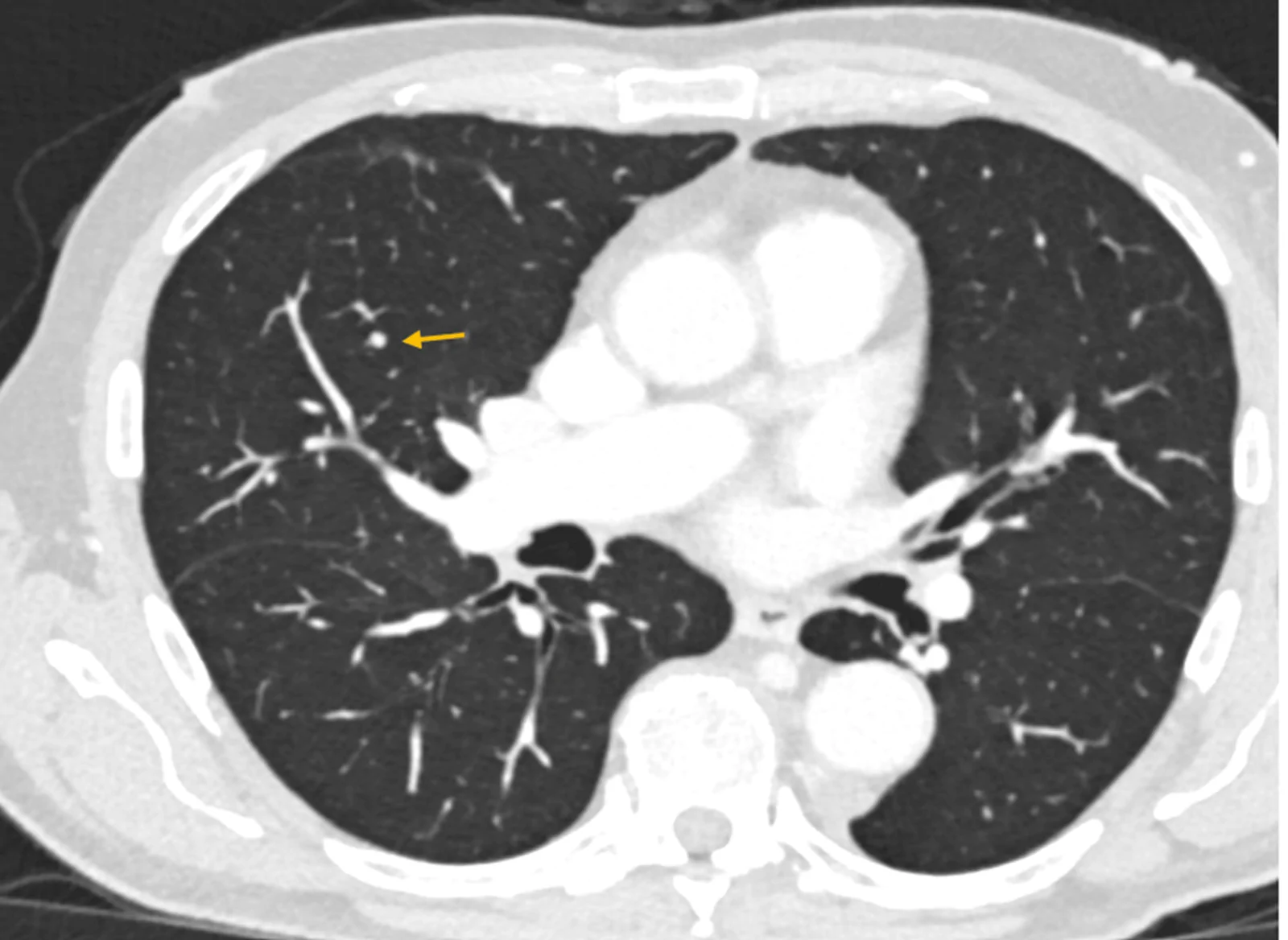
Contrast-enhanced axial CT image showing right lung metastases (yellow arrow).

**Figure 11:**
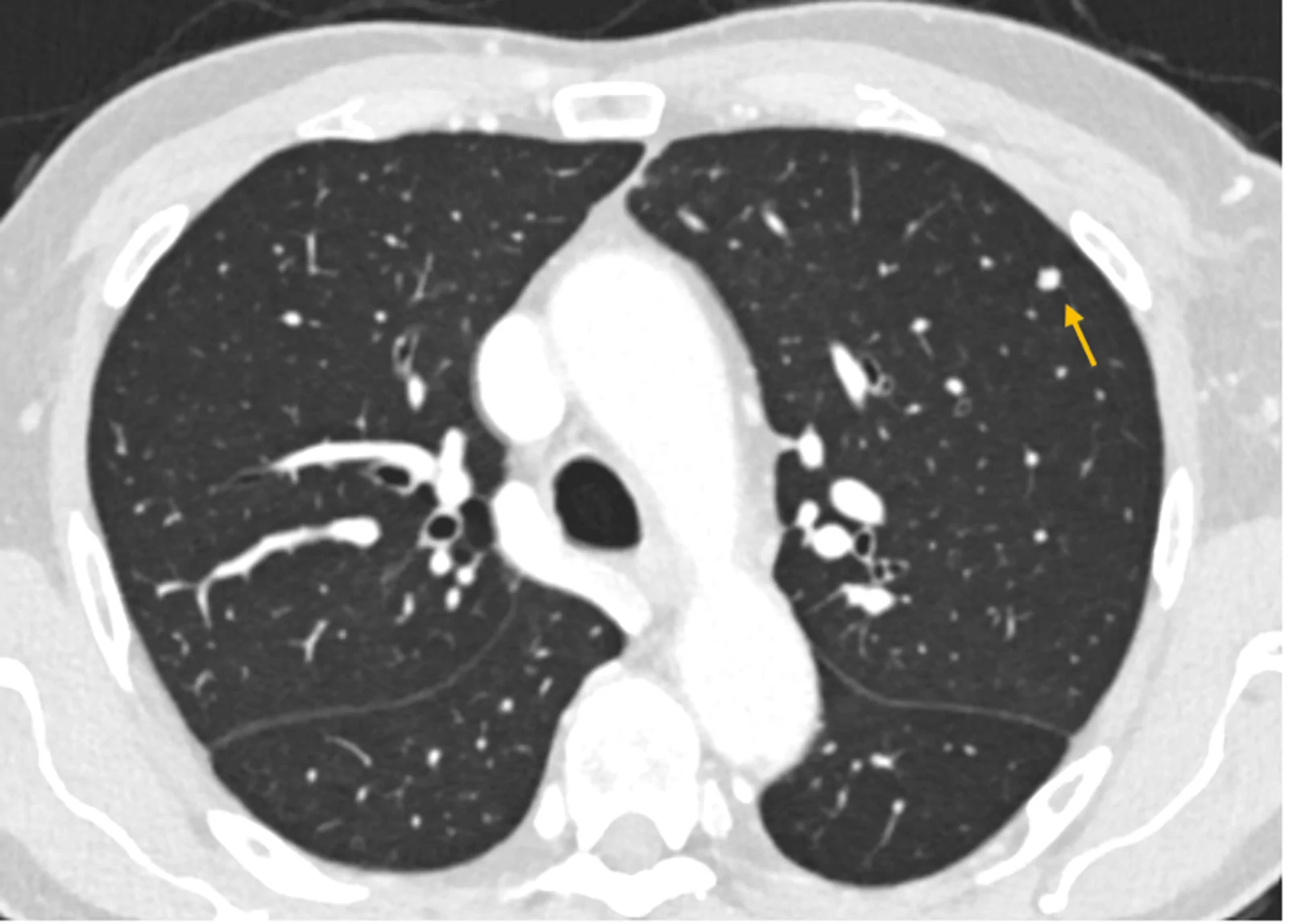
Contrast-enhanced axial CT image showing left lung metastases (yellow arrow).

**Figure 12:**
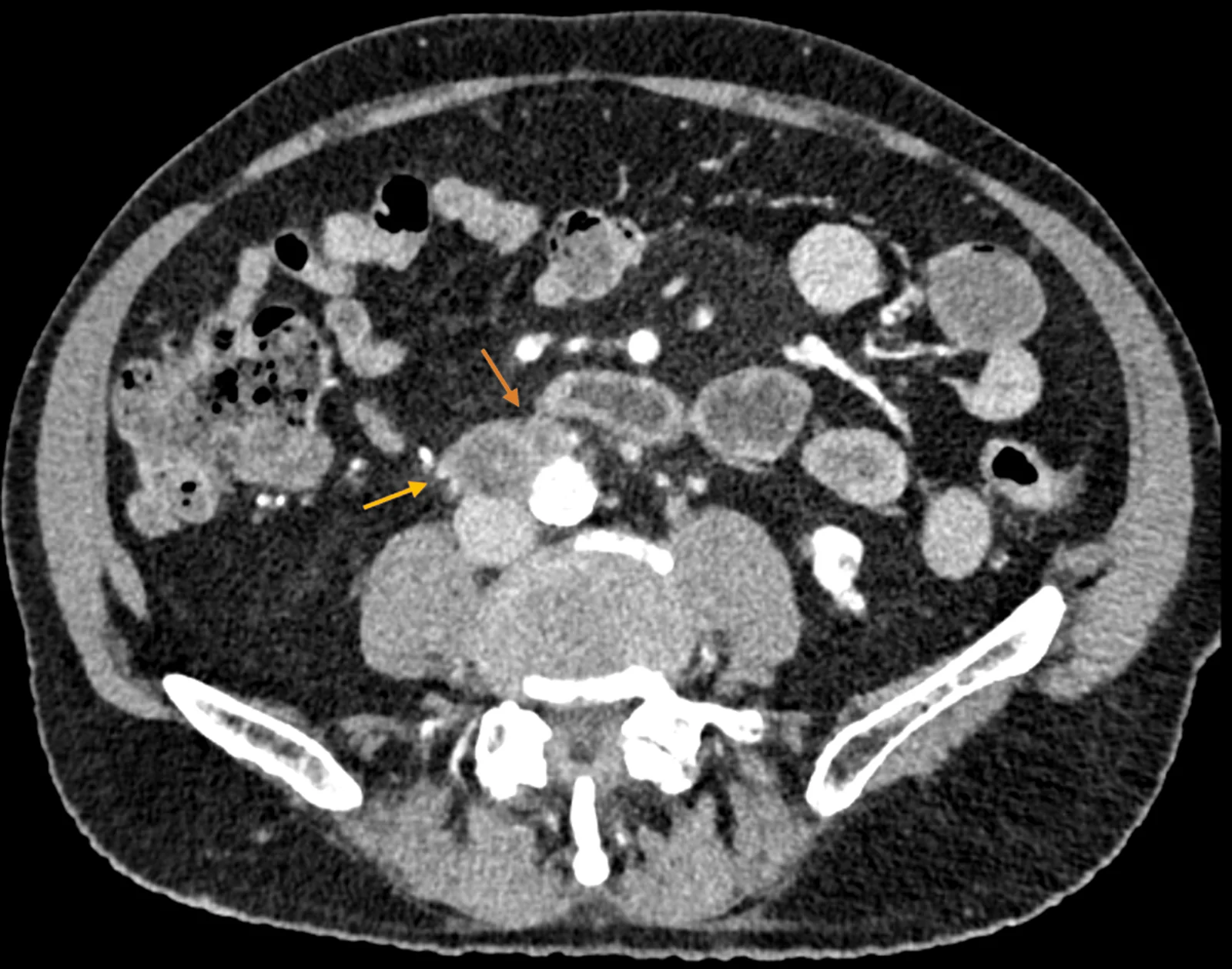
Contrast-enhanced axial CT image demonstrating progressive enlargement of the pre-aortic (orange arrow) and precaval (yellow arrow) lymph nodes.

**Figure 13:**
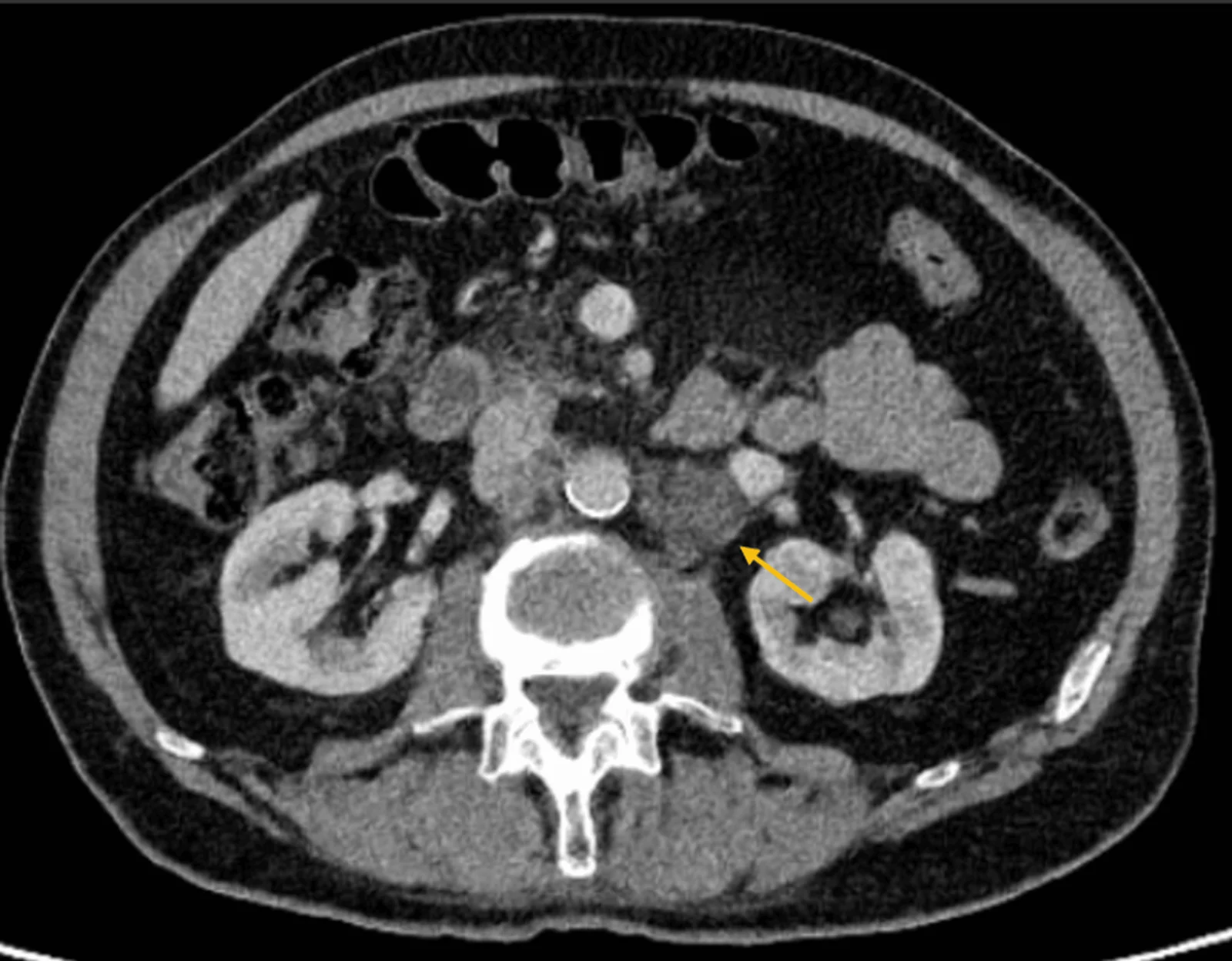
Contrast-enhanced axial CT image demonstrating isolated progression of the left para-aortic lymph node (yellow arrow), consistent with oligoprogression.

Imaging performed in late Year 4 demonstrated further disease progression, with new pulmonary nodules (Figures 10-11) and worsening intra-abdominal lymphadenopathy involving the superior precaval and pre-aortic lymph nodes (Figure 12). Third-line oral vinorelbine (60 mg/m² on Days 1, 8, and 15 of a 21-day cycle) was commenced in late Year 4. After three cycles, imaging performed in early Year 5 demonstrated overall stable disease apart from oligoprogression involving the left para-aortic lymph node (Figure 13). Vinorelbine was continued while the imaging findings were reviewed at the MDT and palliative radiotherapy was arranged, with a total of six cycles administered before radiotherapy. No dose modifications or toxicity-related treatment interruptions were required, and no significant vinorelbine-related toxicity was observed. The patient subsequently received further palliative radiotherapy (15 Gy in five fractions using IMRT) in mid-Year 5. The third radiotherapy field partially overlapped with the previously irradiated intra-abdominal nodal regions, predominantly with the original treatment field. No clinically significant cumulative radiotherapy-related toxicity was observed following the repeated courses of radiotherapy. Vinorelbine was subsequently resumed. At the time of manuscript submission, the patient remained clinically stable with an ECOG performance status of 1 and was awaiting interval CT imaging to assess radiological disease status.

## Discussion

MTVT is an exceedingly rare neoplasm, accounting for less than 1% of all mesotheliomas, with approximately 300 cases described in the literature [1-3]. Although MTVT can occur across a broad age range, it is most frequently diagnosed in older adults [1-3]. Although asbestos exposure has been reported in patients with MTVT, the strength of this association is less clearly established than in pleural mesothelioma [1,3]. Other proposed associations include chronic inflammation, previous scrotal trauma or surgery, and long-standing hydrocele [1-3].

Clinically, MTVT frequently mimics benign scrotal pathology, most commonly presenting as painless scrotal swelling, hydrocele, or a paratesticular mass, resulting in delayed or incidental diagnosis during surgery or histopathological examination [1-4]. In the present case, the patient presented with an apparently uncomplicated hydrocele; however, intraoperative identification of abnormal tunical thickening prompted biopsy and facilitated the diagnosis. This pattern of incidental diagnosis during hydrocele surgery has been repeatedly described in the literature [1,3,4], emphasising the importance of maintaining clinical suspicion even in patients with apparently benign scrotal disease.

Histologically, the epithelioid subtype predominates, while biphasic and sarcomatoid variants are less common [2,3,5]. Tumours typically express mesothelial immunohistochemical markers, including calretinin, cytokeratin 5/6, WT1, and D2-40 [3]. The present case demonstrated epithelioid histology and provides detailed longitudinal clinical and radiological documentation of progression from localised to metastatic disease.

Optimal management of MTVT remains undefined because of its rarity. Radical inguinal orchidectomy with high spermatic-cord ligation is generally regarded as the preferred treatment for localised disease [1-3]. Hemiscrotectomy or wider excision of involved scrotal tissue may be considered following previous trans-scrotal surgery or when local tumour seeding is suspected [1,3]. Despite an apparently localised presentation, recurrence and metastatic dissemination are common, particularly involving the retroperitoneal lymph nodes [1-3]. Older published series have described an aggressive clinical course, with reported median survival generally below three years [1,3]. In our patient, metastatic retroperitoneal lymphadenopathy developed following an initial disease-free interval, consistent with previously described patterns of dissemination.

In the absence of MTVT-specific prospective evidence, systemic treatment strategies for advanced disease are generally extrapolated from pleural mesothelioma. Cisplatin plus pemetrexed demonstrated superior outcomes compared with cisplatin alone in malignant pleural mesothelioma and has consequently informed the use of platinum-pemetrexed combinations in advanced MTVT [3,6]. Carboplatin-pemetrexed may be used as an alternative when cisplatin is considered unsuitable, although evidence specific to MTVT is limited to individual reports and extrapolation from pleural mesothelioma [3]. In the present case, a partial radiological response was observed during carboplatin-pemetrexed chemotherapy after disease progression on immunotherapy.

Immune checkpoint inhibitors have transformed the management of unresectable pleural mesothelioma. The phase III CheckMate 743 trial demonstrated improved overall survival with nivolumab plus ipilimumab compared with platinum-pemetrexed chemotherapy in unresectable malignant pleural mesothelioma [7,8]. However, only isolated case reports have described the use of immunotherapy in MTVT. Mishra et al. described the first reported case of metastatic MTVT showing a partial response to ipilimumab plus nivolumab [9]. In contrast, our patient demonstrated disease progression during dual immune checkpoint inhibition, highlighting the heterogeneity of treatment response and the uncertain efficacy of immunotherapy in this rare subtype. This variability may reflect biological and molecular heterogeneity; however, the determinants of immunotherapy response in MTVT remain poorly characterised.

Evidence supporting radiotherapy in MTVT is limited. Based largely on experience in pleural mesothelioma, radiotherapy may be considered for symptom palliation or local disease control in selected patients [10]. Furthermore, focal radiotherapy has demonstrated feasibility and meaningful local control in selected patients with oligoprogressive pleural mesothelioma [11]. In the present case, repeated courses of radiotherapy were followed by local disease control at the treated sites and periods of overall disease stabilisation. This experience suggests that palliative radiotherapy to sites of oligoprogression may have a role within an individualised multimodal strategy, although its contribution cannot be separated from the effects of systemic treatment.

A notable feature of this case is the heterogeneous response across successive treatment modalities, with progression during dual immune checkpoint inhibition, followed by a partial response during carboplatin-pemetrexed chemotherapy and periods of local disease control following radiotherapy to sites of oligoprogression. This case therefore contributes to the limited literature on metastatic MTVT by describing an individualised, sequential, and multidisciplinary treatment strategy. The periods of disease stabilisation observed over approximately four years of follow-up are particularly notable in the context of the aggressive natural history of MTVT, although conclusions regarding treatment efficacy cannot be drawn from a single case [1,3].

As a single-patient report, this case cannot establish the efficacy of any individual treatment modality or define a standard management strategy. Nevertheless, it provides detailed longitudinal evidence of differential responses to immunotherapy, chemotherapy, and palliative radiotherapy to sites of oligoprogression in advanced malignant mesothelioma of the tunica vaginalis testis. Given the rarity of this malignancy, international case registries, collaborative data collection, and molecular characterisation will be important to clarify its natural history, optimise treatment sequencing, and identify potential predictive biomarkers, particularly for immune checkpoint inhibition.

## Conclusions

This case highlights the importance of multidisciplinary, individualised, and response-adapted management in metastatic malignant mesothelioma of the tunica vaginalis testis. Over approximately four years of follow-up, sequential treatment with immune checkpoint inhibition, platinum-pemetrexed chemotherapy, and palliative radiotherapy to sites of oligoprogression was associated with periods of overall disease stabilisation, while radiotherapy was followed by local disease control at the treated sites. However, the relative contribution of each treatment modality cannot be determined from a single case. Further collaborative research and molecular characterisation are required to clarify the role of immune checkpoint inhibitors, define the potential contribution of palliative radiotherapy to sites of oligoprogression, and optimise treatment sequencing in this exceptionally rare malignancy.
